# *N* ^2^,*N* ^2^,*N* ^4^,*N* ^4^,*N* ^6^,*N* ^6^-Hexapropyl-1,3,5-triazine-2,4,6-triamine

**DOI:** 10.1107/S1600536808025166

**Published:** 2008-08-13

**Authors:** Yu-Feng Li, Fang-Fang Jian

**Affiliations:** a Microscale Science Institute, Weifang University, Weifang 261061, People’s Republic of China

## Abstract

The title compound, C_21_H_42_N_6_, was prepared by the reaction of 2,4,6-trichloro-1,3,5-triazine with dipropyl­amine. The structure of the mol­ecule is tripodal.

## Related literature

For related literature, see: Frassanito *et al.* (1996 ▶); Bishop *et al.* (2002 ▶).
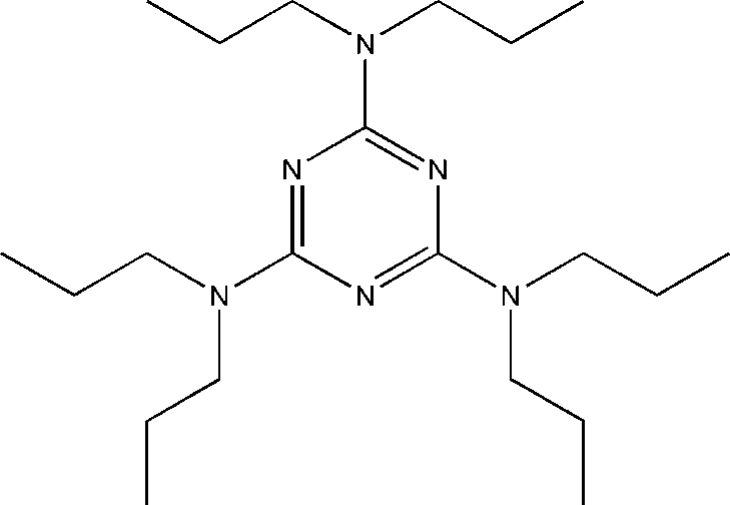


## Experimental

### 

#### Crystal data

C_21_H_42_N_6_
*M* *_r_* = 378.61
Triclinic, 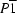

*a* = 9.847 (2) Å
*b* = 12.044 (2) Å
*c* = 12.910 (3) Å
α = 116.57 (2)°
β = 96.94 (4)°
γ = 106.81 (3)°
*V* = 1253.7 (7) Å^3^
*Z* = 2
Mo *K*α radiation
μ = 0.06 mm^−1^
*T* = 295 (2) K
0.32 × 0.24 × 0.13 mm

#### Data collection

Bruker P4 diffractometer
Absorption correction: multi-scan (*DENZO-SMN*; Otwinowski & Minor, 1997 ▶) *T*_min_ = 0.981, *T*_max_ = 0.992
5686 measured reflections
5364 independent reflections
1966 reflections with *I* > 2σ(*I*)
*R*_int_ = 0.019
3 standard reflections
every 100 reflections
intensity decay: none

#### Refinement

*R*[*F* ^2^ > 2σ(*F* ^2^)] = 0.067
*wR*(*F* ^2^) = 0.179
*S* = 1.00
5364 reflections
245 parameters
H-atom parameters constrained
Δρ_max_ = 0.32 e Å^−3^
Δρ_min_ = −0.17 e Å^−3^

### 

Data collection: *XSCANS* (Bruker, 1996 ▶); cell refinement: *XSCANS*; data reduction: *SHELXTL* (Sheldrick, 2008 ▶); program(s) used to solve structure: *SHELXS97* (Sheldrick, 2008 ▶); program(s) used to refine structure: *SHELXL97* (Sheldrick, 2008 ▶); molecular graphics: *SHELXTL*; software used to prepare material for publication: *SHELXTL*.

## Supplementary Material

Crystal structure: contains datablocks global, I. DOI: 10.1107/S1600536808025166/at2607sup1.cif

Structure factors: contains datablocks I. DOI: 10.1107/S1600536808025166/at2607Isup2.hkl

Additional supplementary materials:  crystallographic information; 3D view; checkCIF report

## Figures and Tables

**Fig. 1.**
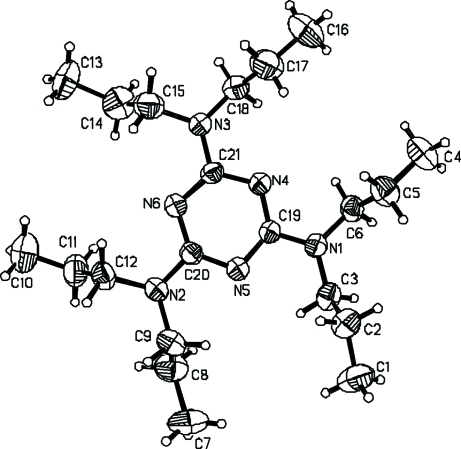
The structure of the title compound showing 30% probability displacement ellipsoids and the atom-numbering scheme.

## supplementary crystallographic information

### Comment

Triazine have received considerable attention in the literature. They are attractive from several points of view, such as the possibility of analytical application (Frassanito *et al.*, 1996). As part of our search for new triazine compounds, we synthesized the title compound (I), and describe its structure here.

In the title compound (I) (Fig. 1), the non-hydrogen atoms of the triazine ring are almost in the same plane, with a maximum deviation of 0.016 (3) Å for C19. The C20—N2 bond length of 1.361 (3)Å is comparable with C—N bond [1.334 (2) Å] reported (Bishop *et al.*, 2002). In the structure, there is no classical hydrogen bonds.

### Experimental

A mixture of the 2,4,6-trichloro-1,3,5-triazine (0.1 mol), and dipropylamine (0.4 mol) was stirred in refluxing ethanol (30 mL) for 5 h to afford the title compound (0.084 mol, yield 84%). Single crystals suitable for X-ray measurements were obtained by recrystallization from ethanol at room temperature.

### Refinement

H atoms were fixed geometrically and allowed to ride on their attached atoms, with C—H distances = 0.96 and 0.97 Å, and with *U*_iso_=1.2–1.5*U*_eq_.

### Crystal data

**Table:**

C_21_H_42_N_6_	*Z* = 2
*M**_r_* = 378.61	*F*_000_ = 420
Triclinic, *P*1	*D*_x_ = 1.003 Mg m^−^^3^
Hall symbol: -P 1	Mo *K*α radiation λ = 0.71073 Å
*a* = 9.847 (2) Å	Cell parameters from 5779 reflections
*b* = 12.044 (2) Å	θ = 1.9–26.8º
*c* = 12.910 (3) Å	µ = 0.06 mm^−^^1^
α = 116.57 (2)º	*T* = 295 (2) K
β = 96.94 (4)º	Prism, colourless
γ = 106.81 (3)º	0.32 × 0.24 × 0.13 mm
*V* = 1253.7 (7) Å^3^	

### Data collection

**Table:**

Bruker P4 diffractometer	*R*_int_ = 0.019
Radiation source: sealed tube	θ_max_ = 27.0º
Monochromator: graphite	θ_min_ = 1.8º
*T* = 295(2) K	*h* = 0→11
ω scans	*k* = −14→14
Absorption correction: multi-scan(DENZO-SMN; Otwinowski & Minor, 1997)	*l* = −15→15
*T*_min_ = 0.981, *T*_max_ = 0.992	3 standard reflections
5686 measured reflections	every 100 reflections
5364 independent reflections	intensity decay: none
1966 reflections with *I* > 2σ(*I*)	

### Refinement

**Table:**

Refinement on *F*^2^	Hydrogen site location: inferred from neighbouring sites
Least-squares matrix: full	H-atom parameters constrained
*R*[*F*^2^ > 2σ(*F*^2^)] = 0.067	1/[σ^2^(*F*_o_^2^) + 0.5*P* + (0.04*P*)^2^ + sinθ/λ], where *P* = 0.5*F*_o_^2^ + 0.5*F*_c_^2^
*wR*(*F*^2^) = 0.179	(Δ/σ)_max_ < 0.001
*S* = 1.00	Δρ_max_ = 0.32 e Å^−^^3^
5364 reflections	Δρ_min_ = −0.17 e Å^−^^3^
245 parameters	Extinction correction: SHELXL97 (Sheldrick, 2008), Fc^*^=kFc[1+0.001xFc^2^λ^3^/sin(2θ)]^-1/4^
Primary atom site location: structure-invariant direct methods	Extinction coefficient: 0.061 (3)
Secondary atom site location: difference Fourier map	

### Special details

**Table:**

Geometry. All e.s.d.'s (except the e.s.d. in the dihedral angle between two l.s. planes) are estimated using the full covariance matrix. The cell e.s.d.'s are taken into account individually in the estimation of e.s.d.'s in distances, angles and torsion angles; correlations between e.s.d.'s in cell parameters are only used when they are defined by crystal symmetry. An approximate (isotropic) treatment of cell e.s.d.'s is used for estimating e.s.d.'s involving l.s. planes.
Refinement. Refinement of *F*^2^ against ALL reflections. The weighted *R*-factor *wR* and goodness of fit *S* are based on *F*^2^, conventional *R*-factors *R* are based on *F*, with *F* set to zero for negative *F*^2^. The threshold expression of *F*^2^ > σ(*F*^2^) is used only for calculating *R*-factors(gt) *etc*. and is not relevant to the choice of reflections for refinement. *R*-factors based on *F*^2^ are statistically about twice as large as those based on *F*, and *R*- factors based on ALL data will be even larger.

### Fractional atomic coordinates and isotropic or equivalent isotropic displacement parameters (Å^2^)

**Table:**

	*x*	*y*	*z*	*U*_iso_*/*U*_eq_	
N1	0.4912 (3)	0.6819 (2)	0.6032 (2)	0.0771 (7)	
N2	0.0405 (3)	0.3446 (2)	0.5011 (2)	0.0786 (7)	
N3	0.1842 (3)	0.4375 (3)	0.2129 (2)	0.0921 (8)	
N4	0.3388 (3)	0.5651 (2)	0.4067 (2)	0.0706 (7)	
N5	0.2667 (3)	0.5137 (2)	0.55636 (19)	0.0705 (6)	
N6	0.1080 (3)	0.3826 (2)	0.3515 (2)	0.0713 (7)	
C1	0.5019 (5)	0.8038 (5)	0.9325 (3)	0.1492 (17)	
H1A	0.4615	0.8624	0.9856	0.224*	
H1B	0.6076	0.8398	0.9664	0.224*	
H1C	0.4595	0.7162	0.9231	0.224*	
C2	0.4656 (4)	0.7929 (4)	0.8097 (3)	0.1069 (11)	
H2A	0.3589	0.7575	0.7757	0.128*	
H2B	0.5069	0.8817	0.8192	0.128*	
C3	0.5281 (3)	0.7023 (3)	0.7252 (3)	0.0900 (10)	
H3A	0.6352	0.7404	0.7584	0.108*	
H3B	0.4909	0.6156	0.7201	0.108*	
C4	0.6951 (4)	0.9729 (4)	0.5583 (4)	0.1302 (14)	
H4A	0.6774	1.0520	0.5724	0.195*	
H4B	0.6883	0.9206	0.4745	0.195*	
H4C	0.7923	0.9987	0.6075	0.195*	
C5	0.5798 (4)	0.8894 (3)	0.5909 (3)	0.1069 (11)	
H5A	0.5856	0.9435	0.6752	0.128*	
H5B	0.4817	0.8650	0.5420	0.128*	
C6	0.6005 (3)	0.7640 (3)	0.5718 (3)	0.0853 (9)	
H6A	0.5946	0.7102	0.4874	0.102*	
H6B	0.6990	0.7888	0.6202	0.102*	
C7	0.1670 (5)	0.3639 (5)	0.8037 (3)	0.1554 (18)	
H7A	0.2222	0.3208	0.8272	0.233*	
H7B	0.0717	0.3407	0.8172	0.233*	
H7C	0.2201	0.4598	0.8510	0.233*	
C8	0.1462 (5)	0.3165 (4)	0.6678 (3)	0.1200 (13)	
H8A	0.2426	0.3380	0.6538	0.144*	
H8B	0.0932	0.2196	0.6200	0.144*	
C9	0.0622 (4)	0.3824 (3)	0.6288 (3)	0.0927 (10)	
H9A	−0.0341	0.3594	0.6426	0.111*	
H9B	0.1146	0.4792	0.6790	0.111*	
C10	−0.2179 (5)	−0.0011 (4)	0.2490 (4)	0.1599 (18)	
H10A	−0.2036	−0.0841	0.2094	0.240*	
H10B	−0.2402	0.0254	0.1912	0.240*	
H10C	−0.2987	−0.0133	0.2830	0.240*	
C11	−0.0829 (4)	0.1031 (4)	0.3453 (3)	0.1207 (13)	
H11A	−0.0013	0.1139	0.3107	0.145*	
H11B	−0.0596	0.0749	0.4024	0.145*	
C12	−0.0979 (3)	0.2375 (3)	0.4121 (3)	0.0880 (9)	
H12A	−0.1298	0.2618	0.3539	0.106*	
H12B	−0.1737	0.2286	0.4526	0.106*	
C13	−0.1129 (5)	0.1115 (4)	−0.0532 (4)	0.1646 (19)	
H13A	−0.1114	0.0240	−0.1008	0.247*	
H13B	−0.1299	0.1473	−0.1045	0.247*	
H13C	−0.1910	0.1045	−0.0157	0.247*	
C14	0.0419 (5)	0.2090 (4)	0.0485 (4)	0.1355 (15)	
H14A	0.0606	0.1727	0.0999	0.163*	
H14B	0.1213	0.2167	0.0113	0.163*	
C15	0.0374 (4)	0.3384 (4)	0.1194 (3)	0.1107 (12)	
H15A	−0.0391	0.3305	0.1594	0.133*	
H15B	0.0122	0.3714	0.0666	0.133*	
C16	0.4074 (6)	0.7152 (5)	0.1645 (5)	0.194 (2)	
H16A	0.3964	0.7963	0.1762	0.291*	
H16B	0.4037	0.6610	0.0818	0.291*	
H16C	0.5010	0.7376	0.2169	0.291*	
C17	0.2822 (5)	0.6369 (4)	0.1941 (4)	0.1364 (15)	
H17A	0.2853	0.6916	0.2775	0.164*	
H17B	0.1875	0.6155	0.1423	0.164*	
C18	0.2973 (4)	0.5110 (3)	0.1757 (3)	0.1099 (12)	
H18A	0.2867	0.4537	0.0909	0.132*	
H18B	0.3955	0.5324	0.2224	0.132*	
C19	0.3614 (3)	0.5835 (3)	0.5187 (3)	0.0672 (7)	
C20	0.1420 (3)	0.4161 (3)	0.4683 (3)	0.0672 (7)	
C21	0.2109 (4)	0.4625 (3)	0.3276 (2)	0.0689 (7)	

### Atomic displacement parameters (Å^2^)

**Table:**

	*U*^11^	*U*^22^	*U*^33^	*U*^12^	*U*^13^	*U*^23^
N1	0.0721 (17)	0.0766 (16)	0.0648 (16)	0.0163 (14)	0.0150 (14)	0.0306 (13)
N2	0.0749 (17)	0.0800 (17)	0.0733 (16)	0.0149 (14)	0.0245 (14)	0.0413 (14)
N3	0.096 (2)	0.0876 (18)	0.0616 (16)	0.0011 (16)	0.0113 (15)	0.0375 (14)
N4	0.0734 (17)	0.0688 (15)	0.0576 (14)	0.0195 (14)	0.0176 (13)	0.0279 (12)
N5	0.0706 (16)	0.0686 (15)	0.0648 (15)	0.0181 (13)	0.0186 (14)	0.0336 (13)
N6	0.0726 (17)	0.0701 (15)	0.0659 (16)	0.0193 (13)	0.0201 (12)	0.0353 (13)
C1	0.172 (4)	0.183 (4)	0.082 (3)	0.065 (4)	0.041 (3)	0.060 (3)
C2	0.108 (3)	0.110 (3)	0.089 (2)	0.042 (2)	0.026 (2)	0.041 (2)
C3	0.074 (2)	0.089 (2)	0.082 (2)	0.0237 (18)	0.0119 (17)	0.0308 (18)
C4	0.128 (3)	0.117 (3)	0.176 (4)	0.043 (3)	0.064 (3)	0.096 (3)
C5	0.110 (3)	0.100 (3)	0.127 (3)	0.045 (2)	0.047 (2)	0.065 (2)
C6	0.064 (2)	0.085 (2)	0.092 (2)	0.0170 (17)	0.0177 (17)	0.0406 (18)
C7	0.208 (5)	0.207 (5)	0.107 (3)	0.106 (4)	0.065 (3)	0.104 (3)
C8	0.142 (4)	0.133 (3)	0.119 (3)	0.072 (3)	0.055 (3)	0.075 (3)
C9	0.090 (2)	0.103 (2)	0.107 (3)	0.039 (2)	0.046 (2)	0.066 (2)
C10	0.134 (4)	0.112 (3)	0.163 (4)	0.019 (3)	0.011 (3)	0.039 (3)
C11	0.099 (3)	0.094 (3)	0.127 (3)	0.010 (2)	0.003 (2)	0.046 (2)
C12	0.074 (2)	0.084 (2)	0.098 (2)	0.0205 (18)	0.0306 (19)	0.045 (2)
C13	0.134 (4)	0.126 (3)	0.122 (3)	0.009 (3)	−0.016 (3)	0.013 (3)
C14	0.142 (4)	0.134 (4)	0.134 (3)	0.065 (3)	0.048 (3)	0.061 (3)
C15	0.143 (4)	0.098 (3)	0.091 (3)	0.041 (3)	0.043 (3)	0.049 (2)
C16	0.199 (5)	0.192 (5)	0.210 (5)	0.022 (4)	0.079 (5)	0.145 (5)
C17	0.135 (4)	0.155 (4)	0.142 (4)	0.049 (3)	0.046 (3)	0.096 (3)
C18	0.144 (3)	0.092 (3)	0.075 (2)	0.026 (2)	0.019 (2)	0.043 (2)
C19	0.071 (2)	0.0604 (17)	0.0604 (18)	0.0230 (16)	0.0166 (16)	0.0252 (15)
C20	0.072 (2)	0.0647 (18)	0.069 (2)	0.0267 (16)	0.0256 (17)	0.0355 (16)
C21	0.080 (2)	0.0698 (18)	0.0536 (17)	0.0290 (17)	0.0182 (16)	0.0295 (15)

### Geometric parameters (Å, °)

**Table:**

N1—C19	1.363 (3)	C7—H7B	0.9600
N1—C3	1.462 (3)	C7—H7C	0.9600
N1—C6	1.466 (3)	C8—C9	1.485 (4)
N2—C20	1.361 (3)	C8—H8A	0.9700
N2—C12	1.456 (4)	C8—H8B	0.9700
N2—C9	1.469 (4)	C9—H9A	0.9700
N3—C21	1.346 (3)	C9—H9B	0.9700
N3—C18	1.481 (4)	C10—C11	1.458 (5)
N3—C15	1.505 (4)	C10—H10A	0.9600
N4—C19	1.339 (3)	C10—H10B	0.9600
N4—C21	1.348 (3)	C10—H10C	0.9600
N5—C20	1.347 (3)	C11—C12	1.518 (4)
N5—C19	1.355 (3)	C11—H11A	0.9700
N6—C21	1.350 (3)	C11—H11B	0.9700
N6—C20	1.343 (3)	C12—H12A	0.9700
C1—C2	1.520 (4)	C12—H12B	0.9700
C1—H1A	0.9600	C13—C14	1.590 (5)
C1—H1B	0.9600	C13—H13A	0.9600
C1—H1C	0.9600	C13—H13B	0.9600
C2—C3	1.499 (4)	C13—H13C	0.9600
C2—H2A	0.9700	C14—C15	1.429 (5)
C2—H2B	0.9700	C14—H14A	0.9700
C3—H3A	0.9700	C14—H14B	0.9700
C3—H3B	0.9700	C15—H15A	0.9700
C4—C5	1.520 (4)	C15—H15B	0.9700
C4—H4A	0.9600	C16—C17	1.522 (5)
C4—H4B	0.9600	C16—H16A	0.9600
C4—H4C	0.9600	C16—H16B	0.9600
C5—C6	1.496 (4)	C16—H16C	0.9600
C5—H5A	0.9700	C17—C18	1.482 (5)
C5—H5B	0.9700	C17—H17A	0.9700
C6—H6A	0.9700	C17—H17B	0.9700
C6—H6B	0.9700	C18—H18A	0.9700
C7—C8	1.548 (4)	C18—H18B	0.9700
C7—H7A	0.9600		
			
C19—N1—C3	120.5 (3)	H9A—C9—H9B	107.6
C19—N1—C6	120.8 (2)	C11—C10—H10A	109.5
C3—N1—C6	118.5 (2)	C11—C10—H10B	109.5
C20—N2—C12	120.5 (2)	H10A—C10—H10B	109.5
C20—N2—C9	120.5 (3)	C11—C10—H10C	109.5
C12—N2—C9	118.8 (2)	H10A—C10—H10C	109.5
C21—N3—C18	120.8 (3)	H10B—C10—H10C	109.5
C21—N3—C15	121.6 (3)	C10—C11—C12	112.3 (3)
C18—N3—C15	117.5 (3)	C10—C11—H11A	109.2
C19—N4—C21	113.6 (2)	C12—C11—H11A	109.2
C20—N5—C19	113.8 (2)	C10—C11—H11B	109.2
C21—N6—C20	113.2 (2)	C12—C11—H11B	109.2
C2—C1—H1A	109.5	H11A—C11—H11B	107.9
C2—C1—H1B	109.5	N2—C12—C11	112.4 (3)
H1A—C1—H1B	109.5	N2—C12—H12A	109.1
C2—C1—H1C	109.5	C11—C12—H12A	109.1
H1A—C1—H1C	109.5	N2—C12—H12B	109.1
H1B—C1—H1C	109.5	C11—C12—H12B	109.1
C3—C2—C1	110.5 (3)	H12A—C12—H12B	107.8
C3—C2—H2A	109.6	C14—C13—H13A	109.5
C1—C2—H2A	109.6	C14—C13—H13B	109.5
C3—C2—H2B	109.6	H13A—C13—H13B	109.5
C1—C2—H2B	109.6	C14—C13—H13C	109.5
H2A—C2—H2B	108.1	H13A—C13—H13C	109.5
N1—C3—C2	113.4 (3)	H13B—C13—H13C	109.5
N1—C3—H3A	108.9	C15—C14—C13	108.8 (4)
C2—C3—H3A	108.9	C15—C14—H14A	109.9
N1—C3—H3B	108.9	C13—C14—H14A	109.9
C2—C3—H3B	108.9	C15—C14—H14B	109.9
H3A—C3—H3B	107.7	C13—C14—H14B	109.9
C5—C4—H4A	109.5	H14A—C14—H14B	108.3
C5—C4—H4B	109.5	C14—C15—N3	111.3 (3)
H4A—C4—H4B	109.5	C14—C15—H15A	109.4
C5—C4—H4C	109.5	N3—C15—H15A	109.4
H4A—C4—H4C	109.5	C14—C15—H15B	109.4
H4B—C4—H4C	109.5	N3—C15—H15B	109.4
C6—C5—C4	112.6 (3)	H15A—C15—H15B	108.0
C6—C5—H5A	109.1	C17—C16—H16A	109.5
C4—C5—H5A	109.1	C17—C16—H16B	109.5
C6—C5—H5B	109.1	H16A—C16—H16B	109.5
C4—C5—H5B	109.1	C17—C16—H16C	109.5
H5A—C5—H5B	107.8	H16A—C16—H16C	109.5
N1—C6—C5	113.8 (3)	H16B—C16—H16C	109.5
N1—C6—H6A	108.8	C18—C17—C16	110.0 (4)
C5—C6—H6A	108.8	C18—C17—H17A	109.7
N1—C6—H6B	108.8	C16—C17—H17A	109.7
C5—C6—H6B	108.8	C18—C17—H17B	109.7
H6A—C6—H6B	107.7	C16—C17—H17B	109.7
C8—C7—H7A	109.5	H17A—C17—H17B	108.2
C8—C7—H7B	109.5	N3—C18—C17	111.5 (3)
H7A—C7—H7B	109.5	N3—C18—H18A	109.3
C8—C7—H7C	109.5	C17—C18—H18A	109.3
H7A—C7—H7C	109.5	N3—C18—H18B	109.3
H7B—C7—H7C	109.5	C17—C18—H18B	109.3
C9—C8—C7	110.8 (3)	H18A—C18—H18B	108.0
C9—C8—H8A	109.5	N4—C19—N5	126.1 (3)
C7—C8—H8A	109.5	N4—C19—N1	117.3 (3)
C9—C8—H8B	109.5	N5—C19—N1	116.6 (3)
C7—C8—H8B	109.5	N5—C20—N6	126.5 (3)
H8A—C8—H8B	108.1	N5—C20—N2	116.6 (3)
N2—C9—C8	114.1 (3)	N6—C20—N2	116.9 (3)
N2—C9—H9A	108.7	N6—C21—N3	117.1 (3)
C8—C9—H9A	108.7	N6—C21—N4	126.8 (3)
N2—C9—H9B	108.7	N3—C21—N4	116.1 (3)
C8—C9—H9B	108.7		
